# Healthy but not RSV-infected lung epithelial cells profoundly inhibit T cell activation

**DOI:** 10.1136/thx.2007.094870

**Published:** 2008-08-18

**Authors:** H Wang, Z Su, J Schwarze

**Affiliations:** 1 Department of Respiratory Medicine, National Heart and Lung Institute and Wright Fleming Institute of Infection and Immunity, Imperial College London, UK; 2 Child Life and Health and Centre for Inflammation Research, University of Edinburgh, UK; 3 Department of Dermatology, First Affiliated Hospital of Nanjing Medical University, China

## Abstract

**Background:**

Respiratory viruses, including respiratory syncytial virus (RSV), can cause asthma exacerbations and bronchiolitis. Both conditions are associated with enhanced cognate immune responses and inflammation and reduced immune regulation. Lung epithelial cells (LECs) can contribute to antiviral and allergic immune responses while gut epithelial cells can inhibit effector T cell responses. A study was performed to determine whether healthy LECs regulate antigen-specific T cell responses and if this regulation is lost during RSV infection.

**Methods:**

LA4 cells, a murine LEC line, infected with RSV or primary murine LECs were co-cultured with ovalbumin-specific T cell receptor transgenic CD4+ T cells from DO11.10 mice and ovalbumin-pulsed bone marrow-derived dendritic cells (DC) to assess T cell proliferation by flow cytometry and cytokine production.

**Results:**

The presence of LECs abrogated DC-induced T cell proliferation and significantly reduced T cell cytokine release. These effects of LECs were predominantly contact-dependent, primarily affected T cells directly and were partly mediated by transforming growth factor β. Soluble factors and DC-mediated effects also contributed to T cell inhibition. RSV infection of LECs reduced their inhibitory capacity in an infection dose-dependent manner. This was independent of proinflammatory cytokines released by infected LECs, but in part due to Toll-like receptor activation and to infection-induced cell death.

**Conclusion:**

Healthy LECs are potent inhibitors of T cell activation, but this regulatory function is lost after RSV infection. These findings suggest a central role for LECs in maintaining the tolerogenic environment of healthy lungs. Loss of this regulatory capacity after viral infection may allow development of excessive cognate immune responses and pulmonary inflammation.

Respiratory viruses, including respiratory syncytial virus (RSV), are the most important triggers of asthma exacerbations.^1^ ^2^ In infants, respiratory viruses can cause severe bronchiolitis^3^ which is associated with an increased risk of asthma development in childhood.^4^ ^5^ Asthma exacerbations and bronchiolitis are thought to be due, at least in part, to reduced immune regulation in the normally tolerogenic environment of the lung and subsequent failure to maintain tolerance to environmental antigens, resulting in excessive and aberrant T cell responses.^6^

The mucosa of the lower respiratory tract, which mainly consists of epithelial cells, provides a physical and functional barrier against inhaled pathogens, allergens and particulates. In respiratory viral infections this barrier is breached and lung epithelial cells (LECs) are the main port of entry for viruses and their main site of replication. LECs are in close contact with a variety of immune cells including antigen-presenting cells such as dendritic cells (DCs) and intraepithelial lymphocytes.^6^ It has recently been recognised that LECs can contribute to antiviral immune responses. Upon viral infection, LECs express type 1 interferons (IFN) which induce antiviral proteins and LEC apoptosis, activate plasmacytoid DCs and promote cellular antiviral responses,^7^ ^8^ as well as proinflammatory cytokines and chemokines. In addition, virus-infected LECs express co-stimulatory molecules which may modulate CD8+ T cell responses.^9^ In asthmatic airways, LECs overexpress interleukin-13 (IL13), a Th2 cytokine that further enhances allergic inflammation and mucus hyperplasia.^10^ In contrast, gut epithelial cells of the colon have been shown to inhibit CD4+ T cell proliferation.^11^ It is not known whether such immune regulatory effects of epithelial cells are unique to the gut or whether they occur in other mucosal sites.

These observations suggest that LECs may be central to both the maintenance of the tolerogenic immune environment of healthy lungs and the switch to inflammation and increased cognate immune responses following respiratory viral infections. We therefore tested the hypothesis that healthy LECs inhibit T cell activation, and that this inhibition is lost in RSV infection.

## METHODS

Detailed information is given in the online supplement.

### Mice

Female BALB/c mice aged 8–10 weeks (Charles River Laboratory, Margate, UK) and DO11.10 mice^12^ (The Jackson Laboratory, Bar Harbor, Maine, USA) were housed under specific pathogen-free conditions and used as sources of bone marrow-derived DCs (BM-DC) and T cell receptor (TCR) transgenic ovalbumin (OVA)-specific CD4+ T cells (DO11.10 T cells), respectively, and under experimental protocols approved by the Home Office, London, UK.

### Virus

Plaque-purified human RSV-A2 (LGC Promochem) and a transgenic RSV strain expressing green fluorescent protein (GFP-RSV)^13^ (Dr M E Peeples, Ohio Sate University) were grown in HEp-2 cells (LGC Promochem, Teddington, Middlesex, UK).

### Generation of BM-DC

Bone marrow cells from femurs were cultured in the presence of recombinant murine granulocyte-macrophage colony stimulating factor (GM-CSF; Life Technologies, Paisley, UK) for 10–12 days, when resultant BM-DC were harvested.^14^

### Lung epithelial cells

LA4 cells, a murine lung alveolar type II epithelial cell line^15^ (LGC Promochem), were grown to confluence in Ham’s F-12 medium (Invitrogen, Paisley, UK).

Primary LECs were isolated from lungs of naïve BALB/c mice by dispase II digestion and subsequent depletion of contaminating mononuclear cells using anti-CD45, anti-CD32/16, anti-CD31 and anti-CD90 antibody and MACS beads (Miltenyi Biotec, Surrey, UK).^16^ Isolated primary LECs were cultured in complete RPMI 1640 medium for 3 days before use in co-culture experiments.

To assess the ability of LECs to inhibit DC-induced T cell proliferation, LA4 cells or primary LECs (2×10^5^/well) were cultured alone for 24 h, when DC/T cell co-cultures were added directly to LECs. In some co-cultures, direct contact of LA4 cells with DCs or T cells was prevented by Transwell chambers (Costar, Fisher Scientific, Loughborough, UK). In antibody neutralisation assays, LA4 cells were incubated prior to and during co-culture with DC/T cells with anti-mouse PD-1 antibody (Clone J43, eBioscience), anti-mouse transforming growth factor β (TGFβ) (Clone 1D11, R&D Systems, Abingdon, UK) or appropriate isotype controls.

In infection experiments, LA4 cells were inoculated with RSV, ultraviolet-inactivated RSV (UV-RSV) or they remained uninfected. After 24 h, LA4 cells were washed and DC/T cells were added. To dissect the effects of RSV on the inhibitory capacity of LECs, LA4 cells were pretreated with thymic stromal lymphopoietin (TSLP), GM-CSF or IL6 (Invitrogen) or Toll-like receptor (TLR) agonists: lipopolysaccharide (LPS) from *E coli* serotype 055:B5, Poly I:C (both from Sigma-Aldrich, Dorset, UK), CpG-ODN1826 (5′-TCCATGACGTTCCTGACGTT-3′) or control ODN1982 (Life Technologies).

### T cell proliferation assays

Following depletion of CD11c+ cells, splenic CD4+ T cells were isolated from DO11.10 mice, both by MACS beads (Miltenyi). Purified DO11.10 T cells (5×10^5^/well) were stained with carboxy fluoroscein succinimidyl ester (CFSE) (Life Technologies), co-cultured with BM-DC (1×10^5^/well) pulsed with OVA or phosphate-buffered saline (PBS) and layered onto LECs or medium. After 4 days, DC/T cells were harvested and reduction in CFSE fluorescence, indicating T cell proliferation, was determined by flow cytometry.^17^ In some experiments proliferation of T cells from BALB/c mice was induced by anti-CD3 antibody and irradiated BM-DC.

### Flow cytometry

Following FC receptor blockade, cells were stained with antibodies to mouse DO 11.10-TCR, CD62L (Caltag Buckingham, UK); Foxp3, GITR, PDL-1 and PDL-2 (all eBioscience, San Diego, California, USA); CD4, CD25, CD3 or isotype controls (all BD Biosciences, Oxford, UK). Samples were acquired using an LSR flow cytometer and CellQuest software (both BD Biosciences) and analysed using WinList software (Verity Software).

### ELISA

Cytokine concentrations in culture supernatants were assessed using the following ELISA kits: mouse GM-CSF, TSLP and TGFβ (all R&D Systems), mouse IL6 and tumour necrosis factor (TNFα) (both Biosource); and antibody pairs: mouse IL1, IL4, IL5, IL10, IL12p70, IL17 and IFNγ with protein standards (all BD Biosciences). OD values at 450 nm were measured by MRXII spectrophotometer (Dynex, Worthing, UK) with Revelation F3.21-software (ThermoBioAnalysis SA, Santa Fe, New Mexico, USA).

### Statistical analysis

Results are expressed as mean (SEM) values. All data were normally distributed (GraphPad Instat) and compared by ANOVA followed by Bonferroni test if p values were significantly different, using GraphPad Prism 4.02 (GraphPad Software). Differences were considered to be significant at p<0.05.

## RESULTS

### Effect of LECs on DC-induced T cell activation

**Figure 1:**
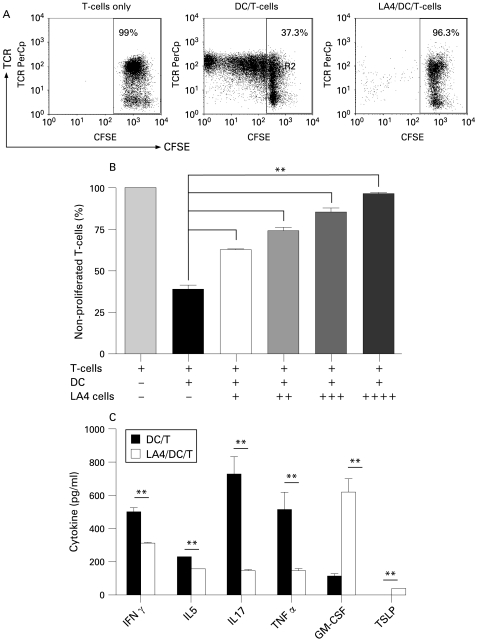
Inhibition of dendritic cell (DC)-induced antigen-specific T cell proliferation and cytokine production by lung epithelial cells (LECs). (A) Carboxy fluoroscein succinimidyl ester (CFSE)-stained DO11.10 T cells were cultured alone or co-cultured with ovalbumin (OVA)-pulsed bone marrow dendritic cells (BM-DC) (ratio 5:1) in the presence or absence of LA4 cells (5×10^5^ cells per well). DC-induced T cell proliferation was assessed by flow cytometry measuring CFSE dilution, shown here in dot plots. The percentages indicate non-proliferated T cells. (B) DC/T cells were co-cultured with increasing numbers of LA4 cells (+: 1×, ++: 2×, +++: 4×, ++++: 6×10^5^ cells per well) and T cell proliferation was assessed and shown as percentages of non-proliferated T cells. (C) Cytokine concentrations in supernatants of these co-cultures were quantified by ELISA. The graphs show mean (SEM) values from a representative experiment (six samples per group) of three independent experiments. Significant differences indicated by horizontal bars; **p<0.01. GM-CSF, granulocyte-macrophage colony stimulating factor; IFNγ, interferon γ; IL, interleukin; TNFα, tumour necrosis factor α; TCR, T cell receptor; TSLP, thymic stromal lymphopoietin.

To assess whether LECs have the ability to influence antigen-specific T cell activation, we analysed CFSE-labelled DO11.10 T cells that were co-cultured with OVA-pulsed BM-DC. After 4 days of culture, strong T cell proliferation was detected. DO11.10 T cells by themselves did not proliferate. If DC and T cells were co-cultured on confluent layers of LA4 cells, the proliferation of DO11.10 T cells was abrogated (fig 1A). Parallel results were obtained with primary murine LECs, which increased the percentage of non-proliferated T cells from 38.09 (3.0)% to 96.01 (0.91)% (see online supplement). This inhibition of T cell proliferation was independent of the DC/T cell ratio used in co-cultures (data not shown), but did depend on LA4 cell numbers seeded at the beginning of culture (fig 1B).

Assessing T cell- and DC-derived cytokines, we found that DO11.10 T cells, BM-DC or LA4 cells cultured alone did not secrete cytokines with the exception of low levels of GM-CSF in LA4 cell cultures (data not shown). In contrast, in DC/T cell co-cultures the T cell cytokines IFNγ, IL5 and IL17 and TNFα were produced (fig 1C). The presence of LA4 cells during DC/T cell co-cultures significantly inhibited production of these cytokines but increased concentrations of GM-CSF and TSLP, which were low or undetectable in the absence of LA4 cells. IL4, IL10 and IL12 were not detected in any of the cultures.

### Contribution of direct cell contact and soluble mediators to T cell inhibition by LECs

**Figure 2:**
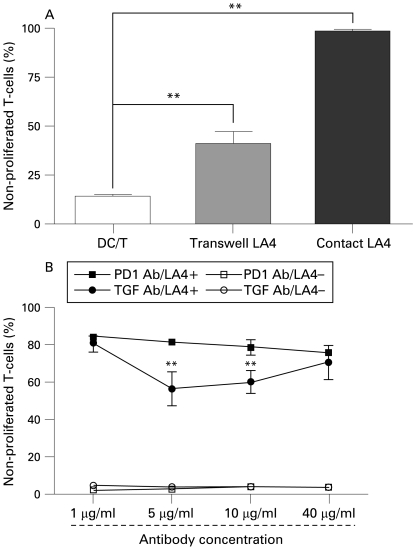
Contribution of direct cell contact and soluble mediators to T cell inhibition by lung epithelial cells (LECs). (A) Dendritic cell (DC)/T cell co-cultures were performed alone, in direct contact with LA4 cells or in transwell plates separating LA4 cells from DCs and T cells. (B) Blocking antibodies to PD-1 and transforming growth factor β (TGFβ) were added at different concentrations to co-cultures of DCs, T cells and LA4 cells. In both experiments, T cell proliferation was assessed after 4 days. The graphs show mean (SEM) percentages of non-proliferated cells from a representative experiment (six samples per group) of three independent experiments. *p<0.05, **p<0.01.

To determine whether the inhibition of T cell proliferation by LECs is dependent on cell contact or mediated by soluble factors, we compared T cell proliferation after co-culture with LA4 cells which were either in direct contact with DC/T cells or separated from them in transwell chambers. Direct cell contact of DC/T cell co-culture with LA4 cells completely prevented T cell proliferation, while co-culture with LA4 cells without direct contact but in the same medium reduced T cell proliferation by about 30% (fig 2A).

Programmed death (PD)-ligand (PDL)-1 and PDL-2 which bind to PD-1 on T cells and TGFβ are all expressed by epithelial cells,^9^ ^18^ as confirmed here (data not shown). These molecules have all been implicated in the inhibition of effector T cell responses.^19^ ^20^ To determine their involvement in LEC-induced T cell inhibition, we used blocking antibodies to PD-1 and TGFβ in LA4 cell/T cell/DC co-cultures (fig 2B). These antibodies did not affect T cell proliferation in control cultures. In the presence of LA4 cells, PD-1 blocking had no effect on LEC-mediated T cell inhibition. In contrast, addition of anti-TGFβ antibody, but not its isotype control (not shown), restored some degree of T cell proliferation.

### Mechanism of T cell inhibition by LECs

**Figure 3:**
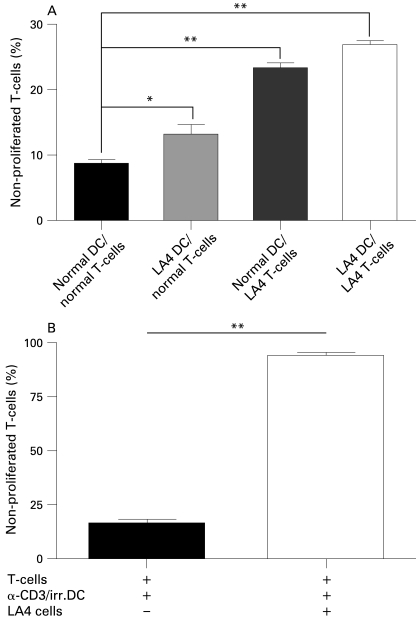
Direct inhibition of T cells by lung epithelial cells (LECs) and, to a lesser extent, via dendritic cells (DCs). (A) Ovalbumin (OVA)-pulsed bone marrow dendritic cells (BM-DC) and carboxy fluoroscein succinimidyl ester (CFSE)-labelled DO11.10 T cells were cultured separately with LA4 cells (LA4 DC, LA4 T cells) or control medium (normal DC, normal T cells) for 24 h. These DC and T cells were then co-cultured in different combinations. (B) Irradiated BM-DC plus antimurine-CD3 antibody were used to induce proliferation of CFSE-labelled splenic CD4+ T cells from naïve BALB/c mice in the presence or absence of LA4 cells. In both experiments, T cell proliferation was measured after 72 h of DC/T cell co-culture. The graphs show mean (SEM) percentages of non-proliferated cells from a representative experiment (six samples per group) of three independent experiments. Significant differences indicated by horizontal bars: *p<0.05, **p<0.01.

The inhibition of DC-induced T cell proliferation by LECs could be a direct effect on T cells or mediated by DCs. To resolve this question, BM-DCs and DO11.10 T cells were separately cultured with LA4 cells for 24 h or exposed to control medium. After removal of LA4 cells, DCs and T cells were co-cultured and T cell proliferation was assessed 3 days later (fig 3A). LEC pretreatment of both DCs and T cells or of T cells alone markedly inhibited T cell proliferation, whereas pretreatment of DCs only with LECs only inhibited T cell proliferation to a small degree. This suggests that T cell inhibition by LECs is primarily due to direct effects on T cells. To confirm this hypothesis, we induced T cell proliferation with anti-CD3 antibody, giving the TCR signal required for activation, and irradiated DCs that provided co-stimulatory signals but were unable to respond to LA4 cells (fig 3B). If LA4 cells were added, the otherwise robust T cell proliferation was prevented almost completely, demonstrating that LECs can directly inhibit T cell activation. This was confirmed using primary LECs (see online supplement).

### Induction of regulatory T cells (Tregs) by LECs in DC/T cell co-cultures

**Figure 4:**
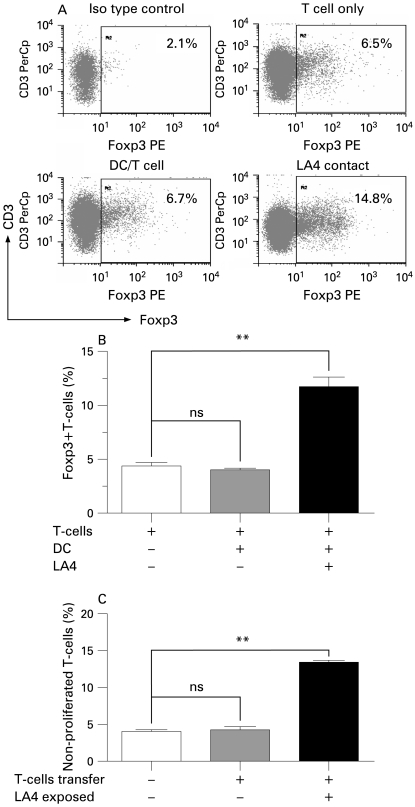
Induction of regulatory T cells (Tregs) in dendritic cell (DC)/T cells co-cultures exposed to lung epithelial cells (LECs). Naïve DO11.10 T cells were co-cultured with ovalbumin (OVA)-pulsed bone marrow dendritic cells (BM-DC) in the presence or absence of LA4 cells. After 48 h of culture, Foxp3 expression in DO11.10 T cells was assessed by intracellular staining and flow cytometry. (A) Dot plots show Foxp3 expression in DO11.10 T cells. (B) Mean (SEM) percentage of Foxp3+ cells of total CD4+ T cells (after subtraction of isotype controls) from a representative experiment of three independent experiments. Significant differences indicated by horizontal brackets: **p<0.01. (C) To determine if LA4 cell-primed T cells inhibit T cell proliferation, T cells were transferred from DC/T cell co-cultures with (LA4 exposed) or without LA4 cells to secondary DC/T cell co-cultures and T cell proliferation was measured in these secondary cultures after 4 days. The graph shows mean (SEM) percentages of non-proliferated cells from a representative experiment (six samples per group) of three independent experiments. Significant differences indicated by horizontal brackets: **p<0.01.

We then investigated possible mechanisms for the lack of T cell proliferation in the presence of LECs. In addition to T cell anergy, induction of regulatory T cells (Tregs) by LECs could inhibit T cell proliferation. We therefore examined the expression of Foxp3, a marker of Tregs, in T cells co-cultured with DCs in the presence or absence of LA4 cells (fig 4A, B). Foxp3 was expressed both in CD25+ and CD25− CD4+ T cells (data not shown), and the level of expression in naïve T cells did not change after stimulation with OVA-pulsed BM-DC. When LA4 cells were added directly to these cultures, the percentage of Foxp3+ DO11.10 T cells trebled. In the absence of direct contact of DC/T cells with LECs in transwell cultures, the induction of Foxp3+ DO11.10 T cells was less pronounced (from 4.5 (0.2)% to 8.1 (0.5)%, p<0.05, n = 6). Furthermore, expression of glucocorticoid-induced tumour necrosis factor receptor (GITR), another marker of Tregs, was increased in T cells after exposure to LA4 cells (see online supplement). These findings indicate that LECs induced cells with a Treg phenotype. To determine whether LEC-exposed T cells, which contain the Foxp3+ population, have immunosuppressive activity, we assessed their influence on T cell proliferation in secondary DC/T cell co-cultures (fig 4C). DO11.10 T cells were stimulated to robust proliferation by BM-DC and this remained unchanged after addition of control T cells from DC/T cell cultures without LECs. In contrast, addition of T cells exposed to LA4 cells significantly inhibited T cell proliferation in secondary cultures, indicating the presence of functional Tregs. In keeping with the inhibitory function of Tregs, CD62L was upregulated in DC/T cell co-cultures in the presence of LECs (see online supplement), indicating suppression of T cell activation.

### Effect of RSV infection of LA4 cells on T cell inhibitory capacity

**Figure 5:**
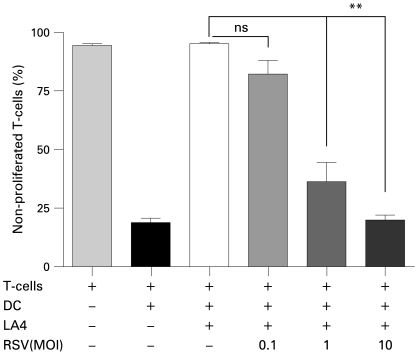
Effect of respiratory syncytial virus (RSV) infection of LA4 cells on T cell inhibitory capacity. 24 h before co-culture with dendritic cell (DC)/T cells, LA4 cells were infected with increasing doses of RSV (MOI = 0.1–10) or with ultraviolet-inactivated RSV as a control. T cell proliferation was determined by carboxy fluoroscein succinimidyl ester (CFSE) assay after 4 days of culture. The graph shows mean (SEM) percentages of non-proliferated cells from a representative experiment (six samples per group) of three independent experiments. Significant differences indicated by horizontal brackets: **p<0.01.

LECs are the primary target for RSV infection in the lower respiratory tract, which leads to enhanced T cell responses and inflammation. We therefore examined whether RSV infection of LECs interferes with their ability to inhibit T cell activation and proliferation. Having ascertained that LA4 cells can be infected with RSV using GFP-RSV (see online supplement), we infected subconfluent LA4 cells with increasing doses of RSV (MOI of 0.1, 1 and 10), added DC/T cell co-cultures to these 24 h later and assessed T cell proliferation after another 72 h (fig 5). Inhibition of T cell proliferation was almost complete in the presence of LA4 cells sham infected with UV-RSV, whereas infection with live RSV decreased LEC-induced inhibition of T cell proliferation in an infection dose-dependent manner. After infection of LA4 cells with RSV at an MOI of 0.1 and 1, T cell proliferation was partially restored and, at an MOI of 10, T cell inhibition was lost completely.

### Effect of TLR-3/-4 activation, RSV-induced cell death and proinflammatory cytokines on T cell inhibitory capacity of LECs

RSV infection in LECs is known to activate TLR-3 through double-stranded RNA and TLR-4 through RSV-F protein, to induce secretion of proinflammatory cytokines and to trigger apoptosis and cell death. To determine whether these factors reduce the ability of LECs to inhibit T cell activation, we initially monitored secretion of proinflammatory cytokines by RSV-infected LECs and found that they had no effect on T cell inhibition by LECs (see online supplement).

**Figure 6:**
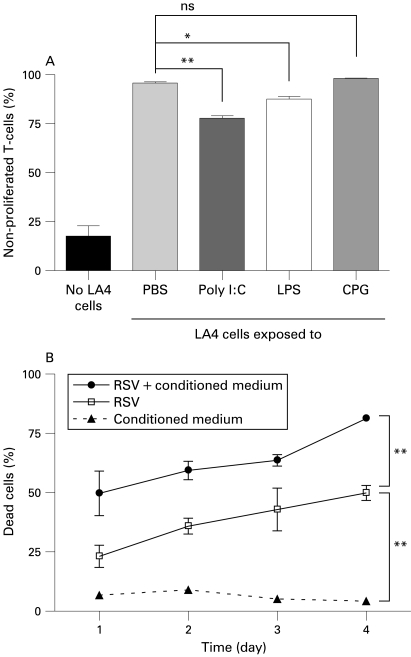
Influence of Toll-like receptor (TLR) ligands and cell death on lung epithelial cell (LEC)-induced T cell inhibition. (A) LA4 cells were preincubated for 24 h with the following TLR ligands: lipopolysaccharide (LPS), poly I:C and CPG1826 (all at 10 μg/ml) or phosphate-buffered saline (PBS) as a control, washed and co-cultured with dendritic cell (DC)/T cells. T cell proliferation was assessed after 72 h by carboxy fluoroscein succinimidyl ester (CFSE) assay. (B) LA4 cells cultured in control medium or conditioned medium from DC/T cell co-cultures were infected with respiratory syncytial virus (RSV) (MOI = 1) and cell viability was monitored over 4 days by trypan blue exclusion. The graphs show mean (SEM) percentages of (A) non-proliferated T cells or (B) percentage of dead cells from a representative experiment (six samples per group) of three independent experiments. Significant differences indicated by horizontal bars (A) or vertical brackets (B): *p<0.05, **p<0.01.

In separate experiments we exposed LA4 cells for 24 h to the TLR-3 ligand poly I:C, the TLR-4 ligand LPS, or to PBS as a control (fig 6A). CpG1826, a ligand of TLR-9 which is not activated by RSV, was used to assess if non-viral TLR activation of LECs also affects their T cell inhibitory capacity. LA4 cells pretreated in this way were then used in co-cultures with DC/T cells and T cell proliferation was assessed after 3 days. While CpG1826 did not have any effect on the ability of LA4 cells to inhibit T cell proliferation, exposure to either LPS or poly I:C significantly reduced this ability and restored some degree of T cell proliferation.

Finally, we investigated if the death of LA4 cells contributes to the reduction in T cell inhibition after RSV infection. In co-cultures with DCs and T cells, LA4 cells formed confluent layers which remained intact up to 48 h after infection and still showed more than 50% confluence at 72 h. To assess cell death by trypan blue exclusion assay over the culture period, LA4 cells were cultured in control medium or in conditioned medium from DC/T cell co-cultures and infected with RSV (MOI = 1) (fig 6B). Without RSV infection, irrespective of the medium used, the percentage of dead cells in LA 4 cultures remained low (<10%) over 4 days. Following RSV infection, LA4 cells cultured in control medium had significantly lower rates of cell death than those cultured with conditioned medium from DC/T cell co-cultures, where the rate of cell death reached 81.29 (1.61)% by day 4. This indicates that LA4 cells are more susceptible to RSV-induced cell death in the presence of mediators secreted by DCs or T cells. The substantial rate of cell death in LECs after infection probably contributed significantly to the reduction in T cell inhibition.

## DISCUSSION

This study aimed to determine whether LECs can inhibit T cell activation and whether RSV infection prevents such an inhibition.

To model antigen-specific T cell responses, naïve DO11.10 T cells were stimulated with OVA-pulsed BM-DC, resulting in robust T cell proliferation.^17^ Murine alveolar type II epithelial LA4 cells^15^ and primary murine LECs were used to represent lower respiratory tract epithelial cells. Co-culture of DC/T cells with confluent layers of LA4 cells or primary LECs abrogated T cell proliferation and significantly reduced T cell cytokine production, indicating that these LECs provided inhibitory signals that prevented normal T cell activation. Such triple co-cultures may well represent healthy lower respiratory tract mucosa which contains T lymphocytes and DCs and provides a tolerogenic environment, minimising inappropriate immune response to harmless inhaled antigens such as allergens.^6^

Delineating the requirements for T cell inhibition by LECs, we found that LECs had to be present early in co-cultures prior to T cell activation, that the extent of T cell inhibition increased with the duration of LEC exposure and that T cell inhibition was not fully reversible after removal of LECs (see online supplement). Transwell cultures preventing direct cell contact of DC/T cells with LECs but not exposure to soluble mediators revealed that direct contact induced almost complete T cell inhibition, while soluble factors only reduced T cell proliferation by 30%. This suggests either that LECs express an inhibitory factor on their surface which is also shed into the medium, or that a combination of independent membrane-bound and secreted factors cause T cell inhibition.

Searching for inhibitory factors, we blocked PD-1 and TGFβ by antibody in triple co-cultures. LECs can express co-stimulatory molecules including PDL-1 and PDL-2.^9^ These bind to PD-1 on T cells, a receptor that provides inhibitory signals and that is implicated in Treg development and T cell tolerance.^21^ Anti-PD-1 treatment did not reduce LEC-induced T cell inhibition, indicating that PDL-1 and PDL-2 are not involved in the process.

TGFβ is secreted by LECs^18^ and is a factor in the induction of some Treg subsets (Th3 cells).^22^ Anti-TGFβ treatment resulted in partial restitution of T cell proliferation, indicating that TGFβ plays a role in T cell inhibition by LECs. Although TGFβ can be secreted and act as a soluble factor, on macrophages and natural Tregs it is primarily active locally on the cell surface.^20^ ^23^ This could explain the strong cell contact-dependent inhibition and the weaker effect of soluble mediators observed here. The limited effect of anti-TGFβ treatment on T cell inhibition may well be due to an inability of the antibody used to neutralise TGFβ secreted locally into areas of cell-cell contact.

To determine if the effects of LECs were directly on T cells or if they were mediated by DCs, T cells or DCs were pre-cultured with LECs prior to DC/T cell co-cultures. In separate experiments, T cells were stimulated with anti-CD3 antibody and irradiated BM-DCs unable to express inhibitory factors de novo upon LEC contact. Both approaches showed that the inhibitory effects of LECs were primarily direct effects on T cells. However, the pre-culture experiments also showed DC-mediated inhibition of T cell activation by LECs. The inhibitory effects of LECs on DCs may explain why DCs from healthy lungs are poor inducers of effector T cell activation.^24^ ^25^

LEC-induced T cell inhibition could be due to clonal deletion, T cell anergy and the induction of Tregs. Clonal deletion is unlikely to play a major role in our model since the numbers of DO11.10 T cells—the majority of which belong to the same transgenic OVA-specific clone—did not decline in the presence of LECs (data not shown). Regulation of T cell responses in many settings is thought to be due to Tregs. Different subsets of Tregs have been described, including naturally occurring Tregs and inducible Tr1 and Th3 cells which mediate immune regulation by IL10 and TGFβ, respectively.^26^ Expression of Foxp3, the critical transcription factor driving Treg development, is a hallmark of naturally occurring Tregs and has also been demonstrated in induced Tregs.^27^ Here, 4–5% of naïve or activated T cells expressed Foxp3 and, in the presence of LECs, the percentage of Foxp3+ T cells trebled, indicating the induction of T cells with a regulatory phenotype. The addition of LEC-exposed T cells from triple co-cultures to secondary DC/T cell co-cultures significantly inhibited T cell proliferation, demonstrating that T cells exposed to LA4 cells did indeed contain functional Tregs. The induction of Tregs by LECs and the finding that LEC-induced T cell inhibition is not completely lost even if LEC contact is ended suggests that, in the healthy lower respiratory tract, Tregs are generated which may retain their immune suppressive effects even if they migrate out of the mucosa to the regional lymph nodes. Here they are likely to contribute to the maintenance of normal immune tolerance to environmental antigens.

Since respiratory viruses are major triggers of airway inflammation and asthma exacerbations, we examined whether viral infection of LECs reduces their immune regulatory function. RSV infection of LA4 cells 24 h before the addition of DC/T cell co-cultures reduced T cell inhibition in a dose-dependent fashion. This effect of RSV infection cannot be explained by accidental infection of DCs, which would have displayed a reduced ability to induce T cell proliferation.^28^

If infection was mimicked by treatment of non-infected LA4 cells with poly I:C (an artificial double-stranded RNA and TLR3 agonist as it occurs during RSV replication) or LPS (a TLR4 agonist like RSV F-protein^29^), T cell inhibition was significantly reduced in subsequent DC/T cell co-cultures. This indicates that some TLR-induced pathways in LECs reduce their ability to inhibit T cell proliferation. In contrast, proinflammatory cytokines from RSV-infected LECs do not change their T cell inhibitory capacity (see online supplement).

Considering that LECs can undergo apoptosis and necrosis during viral infection, we determined whether RSV-induced death of LEC accounts for the reduction in T cell inhibitory capacity. Trypan blue exclusion assays revealed that about 50% of LA4 cells died within 4 days of RSV infection and that DC/T cell-derived mediators rendered them even more susceptible to RSV-induced death, suggesting that the latter contributed significantly to the reduced T cell inhibitory capacity of LECs. This may also apply during natural RSV infection which induces extensive destruction of lower respiratory tract epithelium.^3^ In affected areas normal inhibition of T cells and DCs may be lost, resulting in excessive local T cell responses and subsequent inflammation. This notion is in keeping with the enhanced T cell stimulatory capacity of pulmonary DCs observed following RSV infection^24^ and other respiratory viral infections.^25^ ^30^

Taken together, our findings suggest that LECs inhibit T cell activation in healthy airway mucosa and that they induce Tregs which suppress unwanted adaptive immune responses, not only in the mucosa but also in the associated regional lymph nodes. Upon respiratory viral infection with, for example, RSV, the inhibitory capacity of LECs is compromised, allowing local activation of T cell responses in the respiratory mucosa and consequent airway inflammation. It needs to be recognised that these hypotheses are based on in vitro studies with murine cells. Further studies in human LEC lines and primary LECs from patients with and without viral infection of the lower respiratory tract will be required to validate our findings in the clinical setting.

In conclusion, we report that healthy LECs are potent inhibitors of T cell activation and proliferation and associated cytokine secretion. Our data demonstrate that (1) LECs provide both membrane-bound and soluble inhibitory factors with direct effects on T cells; (2)TGFβ may contribute to this inhibition; and (3) T cell inhibition is, at least in part, achieved through the induction of Tregs. After RSV infection, the T cell inhibitory capacity of LECs is reduced or lost depending on the severity of the infection. This is partly due to TLR activation in LECs and to cell death.
